# Enhancing Data Completeness in Early Detection Pathway of Prostate Cancer: Integration of a Dashboard-Driven Feedback Tool to Improve Quality of Care

**DOI:** 10.3390/jcm13247529

**Published:** 2024-12-11

**Authors:** Lucas C. van Maaren, Nanne Aben, Jolien van Kesteren, Veerle M. D. Struben, Maarten Stals, Kurdo Barwari, Jana Stárková, Erik van Muilekom, Jeroen Visser, Arnoud W. Postema, Matthias F. van Alphen, Marinus J. Hagens, Thierry N. Boellaard, Stijn W. T. P. J. Heijmink, Margriet C. van Dijk-de Haan, Pim J. van Leeuwen, Laura S. Mertens

**Affiliations:** 1 Department of Urology, Netherlands Cancer Institute, 1066 Amsterdam, The Netherlands; l.v.maaren@nki.nl (L.C.v.M.);; 2 Kaiko (Kaiko.AI), Plesmanlaan 121, 1066 Amsterdam, The Netherlands; 3 Department of Intelligence and Analysis, Netherlands Cancer Institute, 1066 Amsterdam, The Netherlands; 4 Department of Radiology, Netherlands Cancer Institute, 1066 Amsterdam, The Netherlands

**Keywords:** prostate cancer, early detection, dashboard, data completeness, quality assurance programs

## Abstract

**Background**: Quality assurance in data collection is essential as data quality directly impacts the accuracy and reliability of outcomes. In the context of early detection of prostate cancer, improving data completeness is a key focus for enhancing patient care. This study aimed to evaluate the effectiveness of a data-driven feedback tool, visualized through a dashboard, in improving the completeness of data collection by healthcare professionals. **Methods**: A cohort of eight healthcare professionals were provided with a dashboard displaying weekly feedback on the completeness of 86 essential data items, including patient demographics, laboratory results, and imaging findings. A comparative analysis of data completeness was conducted for 577 patients enrolled in the prostate cancer early detection pathway, with 211 patients assessed before and 366 patients after the introduction of the dashboard. Statistical analysis was performed using the Mann–Whitney rank-sum test and Chi-square tests. **Results**: The implementation of the dashboard significantly improved data completeness across all healthcare professionals. The average completeness score increased from 0.70 (95% CI 0.67–0.76) before the dashboard’s introduction to 0.88 (95% CI 0.86–0.92) after its implementation, with a *p*-value of <0.001. **Conclusions**: The introduction of a data-driven feedback dashboard significantly enhanced data completeness within the prostate cancer early detection pathway. This improvement has the potential to positively impact the quality of care and to support the generation of high-quality data for future research.

## 1. Introduction

Quality assurance programs have become integral to data-driven healthcare initiatives, guiding improvements and maintaining healthcare quality and outcomes. With the transition towards electronic health records, integrating real-world data into day-to-day clinical practice presents new opportunities. This integration facilitates the collection of efficient and relevant data that can support healthcare professionals in both clinical practice and scientific research.

These quality assurance programs have proven especially valuable within standardized care settings with predefined data points, such as in cancer care pathways. For example, the integration of prostate-specific antigen (PSA) testing, risk calculators, and MRI scans has led to standardized pathways for early detection of prostate cancer, streamlining information gathering and reducing unnecessary biopsies and overdiagnosis [1,2,3,4,5,6,7,8]. By incorporating PSA testing, risk calculators, and MRI scans into risk assessments, the benefit–harm ratio in early detection of prostate cancer is expected to improve. Continuous and direct feedback of real-world data is crucial for evaluating and refining risk calculators and optimizing the quality of care. Additionally, this approach of continuous feedback not only enhances immediate clinical outcomes but also propels healthcare into a new era founded of real-world data-driven science, thereby enhancing prospective research [9].

Data completeness is critical in the transition from paper to electronic health records. Establishing standards for achieving data completeness remains challenging [10,11]. To enhance data completeness, meticulous recording of all clinical parameters and outcomes in prospective registry databases is essential [12]. Adhering to the longstanding principle of ‘garbage in, garbage out’, key elements for meaningful analyses include data accuracy, reliability, and completeness [13]. Previous research has explored the impact of data-driven dashboards on various aspects, including clinical decision making, task completion time, satisfaction, research, and adherence to clinical guidelines, thereby strengthening quality assurance programs. However, the potential of utilizing a dashboard to provide a straightforward solution for data completeness has not been thoroughly investigated.

Therefore, in this study, we focus on the utilization of a data-driven dashboard feedback tool to specifically improve data completeness. This study is situated at a national comprehensive cancer center with a dedicated clinic for early detection of prostate cancer. We aim to assess whether the implementation of a data-driven dashboard feedback tool, focused on predefined data items within this pathway, can effectively improve data completeness. Consequently, we aim to advance healthcare into a real-time data-driven science and thereby facilitate prospective research initiatives towards enhanced quality of care.

## 2. Materials and Methods

### 2.1. Study Design

This quantitative clinical data registry study was conducted at the Center for Early Detection of the Netherlands Cancer Institute (NCI). The institutional review board approved this study and waived the requirement for informed consent (IRBd19-248). We included all men enrolled in the early detection pathway for prostate cancer between April 2022 and August 2023 based on the following criteria: age over 40 years, abnormal PSA level, and a life expectancy greater than 10 years.

### 2.2. Pathway

**Figure 1:**
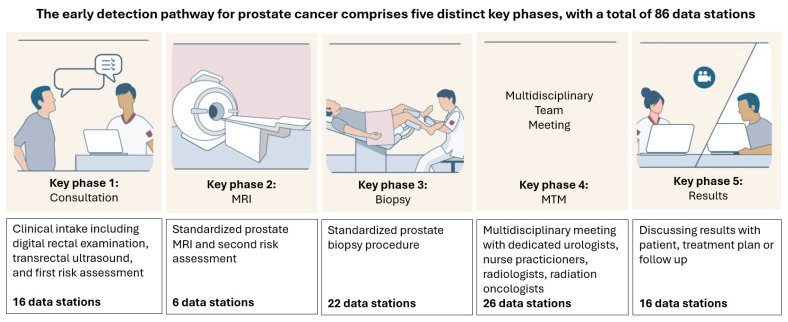
The early detection pathway for prostate cancer comprises five distinct key phases, with a total of 86 data stations collected throughout. Specifically, Phase 1 includes 16 data stations, Phase 2 includes 6, Phase 3 includes 22, Phase 4 includes 26, and Phase 5 includes 16 data stations. All patients progress through Phases 1 and 2. Based on the outcomes in these initial phases, patients are subsequently enrolled in either Phases 3 to 5 or, if prostate biopsies are not performed, in Phases 4 and 5.

The early detection pathway for prostate cancer at the Center for Early Diagnosis of the Netherlands Cancer Institute consists of five distinct key phases (Figure 1). Patients were seen by a team of experienced healthcare professionals, comprising three urologists and five specialized nurse practitioners, each with over 24 months of experience in their current roles.

#### 2.2.1. Phases of the Pathway

In brief, Phase 1 includes anamnesis, medical prehistory, family history, physical examination, transrectal ultrasound (TRUS), and risk assessment [14]. This is followed by prostate MRI, with MRI findings discussed in a second consultation on the same day, along with a reassessment of the patient’s risk for prostate cancer (Phase 2). This reassessment determines whether a prostate biopsy should be performed [2].

Patients with MRI-suggestive lesion for prostate cancer (PI-RADS classification score ≥ 4) or PI-RADS classification score 3 and a PSA density ≥ 0.15 ng/mL underwent targeted and perilesional prostate biopsies, as per protocol (Phase 3). Each consultation, including biopsies if required, was reviewed and registered twice weekly in a multidisciplinary team meeting (MTM), involving urologists, radiotherapists, radiologists, and specialized nurse practitioners (Phase 4). Finally, the prostate biopsy results and the corresponding management plan, or follow-up advice if no biopsies were performed, were communicated to all individuals during a physical or video consultation one week after the initial consultation.

All patients progressed through Phases 1 and 2, and depending on the outcomes, they were subsequently enrolled in Phases 3 to 5 or in Phases 4 to 5 if prostate biopsies were not performed.

#### 2.2.2. MRI Evaluation and Biopsy Strategy

All MRI scans were evaluated by experienced, dedicated prostate cancer radiologists with over 5 years of experience in prostate MRI interpretation. The standardized five-point ‘Prostate Imaging Reporting and Data System’ (PI-RADS) classification was used, in concordance with the PI-RADS version 2.1 guidelines [15]. Transperineal prostate biopsy procedures for PI-RADS lesions were conducted using MIM image fusion software (Version 7, MIM Software INC., Cleveland, OH, USA) and performed by experienced urologists [16]. Biopsies were taken under local anesthesia with a spring-loaded biopsy gun with an 18 G needle, and no antibiotic prophylaxis was administered. Clinically significant prostate cancer was defined as Gleason score ≥ 3 + 4, equivalent to International Society of Urological Pathology (ISUP) grade group of ≥ 2 [17].

### 2.3. Dashboard Implementation

The introduction of the early detection pathway of prostate cancer at our center involved the immediate establishment of an integrated file encompassing all data stations for patient registration across the different phases. Following a familiarization period, healthcare professionals became used to registering patient data as part of routine clinical practice. When we decided to implement a dashboard to monitor data completeness, a one-month wash-in period was conducted to prepare for its definitive use. Weekly dashboard feedback provided information on data completeness by tracking completeness over time for each key phase and healthcare professional. The initial objective was to use and evaluate the dashboard over a three-month period.

### 2.4. Data Stations

The dashboard incorporated a total of 86 essential data items (Table S1). These data items were coded as ‘stations’, which were spread over five key phases and were integrated into a robust electronic dashboard developed by professional data analysts to meet quality requirements [18]. An innovative audit process was devised to facilitate real-time data entry across the five distinct key phases, using an integrated file consisting of multiple pre-ordered options, risk calculators, measurements, and open text.

Phase 1 consisted of 16 stations, Phase 2 had 6 stations, Phase 3 had 22 stations, Phase 4 had 26 stations, and Phase 5 had 16 stations. These stations included information on, e.g., laboratory results, psychical examinations, co-morbidity, family history, quality of life assessments, MRI outcomes, and biopsy outcomes.

### 2.5. Data Completeness

**Figure 2:**
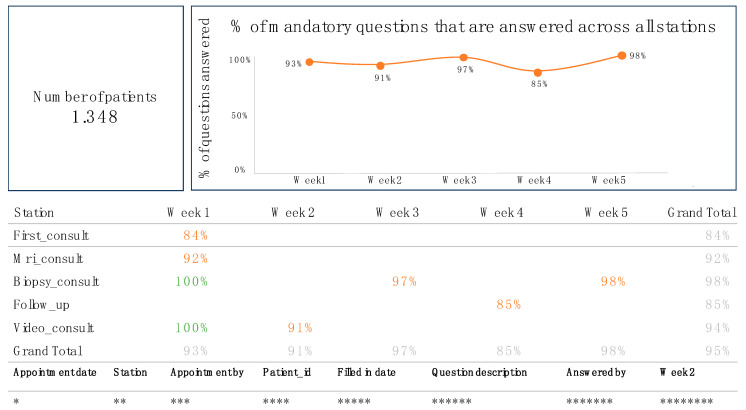
The overall completeness dashboard is shown, with the first column (‘station’) displaying the five key phases (first consult, MRI consult, biopsy consult, follow-up, and video consult). The subsequent columns present data completeness across these phases over five consecutive weeks (Week 1 to Week 5), followed by a total completeness score (‘Grand total’). The colors of the percentages provide visual feedback, with 100% displayed in green, 80–99% in orange, and below 80% in red (not applicable in Figure 2). By clicking on the percentages, detailed information about the following areas is displayed: *: date of appointment; **: name of data station; ***: name of healthcare professional for the appointment; ****: patient identification number; *****: date of data entry; ******: description of the question (see Supplementary S1); *******: name of healthcare professional who entered the data; and ********: results of the question.

Data completeness was measured as the percentage of successful data stations by healthcare professionals for each patient during specific key phases. Any inconsistencies between reported data in electronic health records and extracted data in the dashboard were discussed between the data analysts and healthcare professionals. The dashboard provided a comprehensive virtual display of individual and average completeness scores, as shown in Figure 2. Results were presented as percentages for each healthcare professional and included graphical depictions of performance. Detailed information for each data station could be accessed by clicking on the percentages displayed in the dashboard, revealing specifics such as appointment date, station, appointment by, patient ID, date, filled-in date, question description, answered by, and week.

### 2.6. Feedback

**Figure 3:**
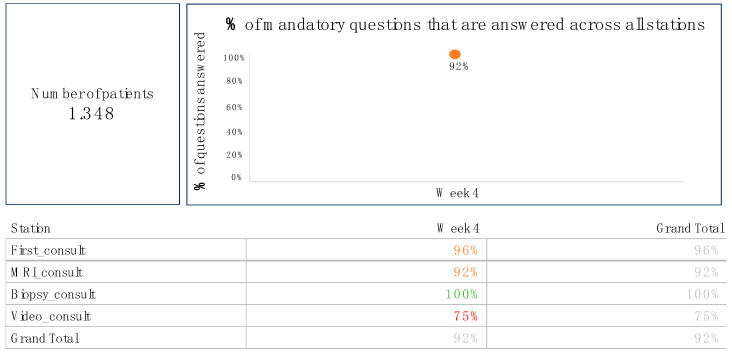
Example of data-driven dashboard feedback on data completeness, received weekly by each healthcare professional. The first column displays the phases first consult, MRI consult, biopsy consult, follow-up, and video consult), while the subsequent columns show data completeness across these phases for a specific healthcare professional during a given week. Percentages are color-coded for visual feedback: 100% is shown in green, 80–99% in orange, and below 80% in red.

Each healthcare professional received weekly feedback on their data entry completeness via secure professional email, highlighting their individual score as a percentage and compared to the group average. An example of the weekly data-driven dashboard feedback is shown in Figure 3. A designated user (PvL) and a quality officer from the Early Diagnosis Center monitored scores for all team members, encouraging improvement when scores fell below 90%. There was no mandatory protocol for corrective active.

### 2.7. Questionnaire

To evaluate the experience of healthcare professionals with the dashboard implementation within the care pathway, we administered a questionnaire consisting of ten items [19,20], focusing on the following five key domains: usefulness, interface, reliability, effectiveness, and adaptation. Within each domain, two items were developed, using a 5-point Likert-type agree–disagree scale. All eight healthcare professionals were invited to complete this questionnaire digitally. Table 1 in the results section provides a detailed list of the questionnaire items.

### 2.8. The Literature Review

To support our findings with existing knowledge, we conducted a literature review on the implementation of data dashboards within healthcare settings (Table 2). This review aimed to provide context for our results and identify similar applications of dashboards focused on enhancing data completeness. By examining previous studies, we sought to understand the effectiveness and challenges of dashboard tools in improving data quality and clinical workflows, thereby reinforcing the relevance of our approach.

### 2.9. Statistical Analyses

All clinical data were recorded in electronic health records using the standard system (HiX, version 6.3, Chipsoft B.V., Amsterdam, The Netherlands). Categorical completeness data were summarized as frequencies and percentages, while continuous variables were presented as medians with interquartile ranges (IQRs). Group comparisons were conducted using the Mann–Whitney rank-sum test for continuous variables and the Chi-square test for categorical variables.

A quantitative analysis of data completeness was performed by comparing data completeness before and three months after the dashboard implementation, following a one-month wash-in period. Completeness was assessed for each individual healthcare professional as well as collectively for all healthcare professionals at each phase of the pathway, expressed in frequencies and percentages.

The Student’s *t*-test was used to compare data entry completeness before and after the introduction of the data-driven dashboard feedback tool. All statistical tests were two-sided, with significance defined as *p* < 0.05. Statistical analyses were conducted using IBM SPSS Statistics for Windows, Version 22.0.

Additional long-term analyses at 6 and 12 months are planned to evaluate the lasting effects of the dashboard intervention.

## 3. Results

### 3.1. Patient Characteristics

In total, 577 participants were included in the study, with 211 patients enrolled before the introduction of the dashboard and 366 patients after its implementation. Patient characteristics are shown in Table 3. The participants had a median age of 67 years (IQR 61–73) and a median PSA value of 7.9 ng/mL (IQR 4.7–12.4). Among the participants, 230 (39.9%) had an abnormal direct rectal exam (DRE) and 101 (17.5%) had previously undergone a negative prostate biopsy.

All participants underwent a prostate MRI, with findings suggestive of prostate cancer in 389 (67.4%) men. Specifically, 211 (36.6%) men had a PI-RADS classification score of five, 133 (23.1%) had a score of four, and 45 men had a score of three with a PSA density ≥ 0.15 ng/mL, leading to subsequent prostate biopsies. Among the 389 participants with an indication for prostate biopsy, prostate cancer was detected in 304 (78.1%) men, with clinically significant prostate cancer (ISUP ≥ 2) identified in 230 men (59.1%).

For those with a PI-RADS score of three, prostate cancer was detected in 17 men (17.1%), with clinically significant cancer (ISUP ≥ 2) in 8 men (8.1%). For the 133 men with a PI-RADS score of four on MRI, prostate cancer was detected in 94 (70.7%) men and clinically significant cancer (ISUP ≥ 2) was detected in 65 men (48.9%). Among the 211 men with a PI-RADS score of five on MRI, prostate cancer was detected in 189 (89.6%), with clinically significant cancer (ISUP ≥ 2) found in 157 men (74.4%).

### 3.2. Completeness Dashboard

Prior to implementing the data-driven dashboard feedback tool, the overall data entry completeness in the electronic health records was 70%. The initial average completeness score for the three urologists was 85%, while for the nurse practitioners, it was 64%.

**Figure 4:**
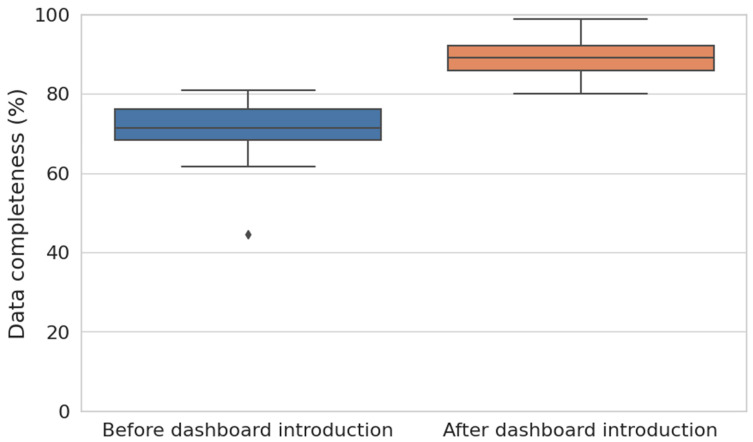
Boxplots showing a statistically significant improvement in overall data completeness scores over a three-month period. The blue boxplot represents the data completeness scores before the introduction of the data-driven dashboard feedback tool, while the orange boxplot shows the scores after its implementation. This figure highlights the increase in completeness following the intervention, illustrating the tool’s positive impact on data entry practices among healthcare professionals.

Following implementation, completeness scores improved across all phases for all healthcare professionals, with the average data completeness rising from 70% (95%CI 67–76) to 88% (95%CI 86–92), *p* < 0.001 (Figure 4). Specifically, the overall completeness scores increased among urologists, with the average completeness rising from 85% to 92%, *p* = 0.03. Among specialized nurse practitioners, the average data completeness increased from 64% to 88, *p* < 0.001.

Due to the limited number of healthcare providers involved, no additional analyses could be conducted regarding the influence of role, gender, age, or experience on data completeness outcomes.

### 3.3. Questionnaire Responses

Responses from all eight healthcare professionals were collected, achieving a response rate of 100%. The overall median score of the questionnaire was 4.3 (IQR 3.7–4.7), reflecting a positive evaluation of the dashboard (Table 1). Notably, three items received a median score of five, including both items within the domain of effectiveness. Conversely, three items had an interquartile range (IQR) beginning at three, with two of these items belonging to the domain of reliability.

## 4. Discussion

In this study, we evaluated the impact of implementing a data-driven dashboard feedback tool on data completeness within a specialized pathway for the early detection of prostate cancer at a dedicated center for early detection embedded in a national cancer institute. Our findings show a significant improvement in data completeness with the dashboard tool, suggesting that active engagement of healthcare professionals is critical in enhancing data completeness and data quality within quality assurance programs. The direct improvement in data completeness following the intervention emphasizes the importance of real-time feedback, which raises awareness among healthcare providers, strengthens intention, and facilitates practical changes in daily practice, particularly concerning data quality.

The implementation of the data-driven dashboard feedback tool led to improved data completeness among both urologists and specialized nurse practitioners in our early detection pathway. Improvements were observed across all phases of the pathway, indicating that the dashboard can be efficiently used across diverse professional roles and could be applied to other clinical settings.

Our literature review (Table 2) contextualizes these findings, as dashboards have consistently proven effective in enhancing data completeness and improving clinical workflows. We observed a considerable improvement in data completeness, consistent with findings from similar studies where dashboards supported patient safety, streamlined workflows, and guided clinical decisions. However, to the best of our knowledge, this study is one of the first to combine the implementation of a dashboard with real-time feedback to enhance healthcare professionals’ performance of data completeness within an oncologic setting of a prostate cancer early detection pathway, an approach aligned with the innovative model of a dedicated cancer early detection center.

Although data completeness is just one element of a broader quality cycle in early prostate cancer detection [21,22], complete data support better-informed clinical decisions [23]. While our study primarily focuses on data completeness, the ultimate goal is to facilitate clinical benefits such as more accurate diagnoses and treatment decisions. Future work will address the dashboard’s long-term impact on clinical outcomes, including how data completeness might influence multidisciplinary team recommendations, biopsy timing, and other aspects of patient management. These results will be explored in follow-up studies.

A limitation of this study is its focus on short-term improvements in data completeness, leaving the long-term sustainability of these effects unknown. We plan to collect follow-up data at six and twelve months to assess the dashboard’s lasting impact. Longitudinal data, as well as evaluating the dashboard across multiple centers, would also allow us to explore potential differences in dashboard effectiveness among healthcare professionals. The present analysis is limited by a relatively small sample size, although it includes all involved healthcare professionals at our center.

Our study’s strength lies in integrating the expertise of urologists and specialized nurse practitioners within a standardized oncologic pathway. This approach contrasts with recent studies, which seldom focused on nurse-sensitive indicators [36], despite their suitability for standardized care pathways. Notably, we observed baseline differences in data completeness, with urologists showing higher initial scores than nurse practitioners. This disparity may reflect experience, role-based factors, or even skepticism about data entry practices. Due to our sample size, we could not conduct subgroup analyses based on these characteristics, but future studies with larger sample sizes could explore such influences. Additionally, incorporating a qualitative component in future studies, such as semi-structured interviews, would allow us to identify specific barriers impacting data completeness among different professional groups and inform strategies to optimize data entry as well as dashboard engagement.

Questionnaire results indicate an overall positive reception, especially regarding effectiveness, suggesting successful behavior change among healthcare professionals. However, the lowest scores were reported in reliability, reflecting concerns regarding data entry consistency. To address this, we recommend exploring automated error-checking mechanisms and real-time alerts for missing or inconsistent data in future iterations. Tools such as pop-up notifications or “go/no-go” requirements before advancing in the pathway could prevent data entry gaps and enhance reliability. While these features could improve reliability scores, they may impact usability, as reflected in the interface domain. Additionally, although the binary and pre-ordered options for data entry improved completeness, they may limit the capture of nuanced clinical information, potentially impacting data validity. Future iterations should consider balancing standardized fields with open-text options to preserve data quality.

This study underscores that dashboards are increasingly used in healthcare settings to visualize data and improve the quality of care. Lessons from previous studies show that dashboards meet various requirements for enhancing data quality, including implementation processes that involve healthcare professionals at each stage. We expect that once the improvement in data registry among healthcare professional succeeds, it will become integrated into the weekly routine as the new standard. This expectation aligns with findings from a systematic review underlining factors contributing to sustained behavior adaption, where structural reinforcement, reflected in our study by the weekly dashboard tool, and effective outcomes, such as the visualization of improvement of data completeness, contribute to maintaining behavior change. Continuous repetition, integral in this pathway, is a key factor in habit formation [37].

Our results also suggest that incorporating a data-driven dashboard can effectively improve data completeness in oncologic pathways and involving healthcare professionals throughout its development is crucial for successful integration. This is in line with findings from previous pieces of literature (Table 2). For example, a systematic review including 33 publications on patient safety dashboards presented a growing use of the tool over the past 15 years. However, information regarding the direct impact of dashboards and generalizability was limited, and future assessments were recommended to explore its potential to support patient safety monitoring [24]. Furthermore, an intensive care unit dashboard visualizing and monitoring care of critically ill patients during the COVID-19 pandemic was found to be useful and positively impacted clinician workflows, whereas its adaption into clinical practice was limited [25].

Another example is a recent observational study obtaining data through mobile and wearable devices of patients with Parkinson’s disease in a home setting, which were visualized in a data-driven dashboard and reviewed by clinicians over a six-month period. Clinicians provided feedback leading to ten updates during the study, demonstrating the feasibility of translating data from an app to a dashboard [27]. In another study, a web-based dashboard was developed to provide real-time feedback to residents using various assessment metrics derived from multiple data sources to ease the burden of manual documentation and provide residents with an overview of their educational experience [28]. Additionally, a study assessed the technical performances of surgeons using sensitized tools incorporated into a data-driven dashboard to establish comparisons between expert surgeons and to develop artificial intelligence models [29]. Interestingly, a recent study focused on improving healthcare spending, specifically on laboratory utilization in a tertiary academic medical center, through a weekly performance feedback report. The intervention resulted in a decrease in laboratory test utilization, perpetuating a promising intervention tool to improve the quality of care [30]. Finally, a qualitative study engaged healthcare professionals in the development of quality and safety dashboards to improve usability in clinical practice, proposing a five-stage process [28]. These previous studies show that the use of data-driven dashboards in healthcare setting has grown rapidly over the past years.

An important issue is that the significant increase in data completeness observed with the feedback tool suggests that data entry errors or omissions were a major contributor to data incompleteness in this pathway. These real-life data are essential for improving the development of personalized risk calculators and the accurate interpretation of MRI imaging results, thereby influencing decision making regarding prostate biopsies. These applications appear to be readily transferable to other oncological and non-oncological healthcare pathways. From a scientific perspective, it is important to acknowledge that statistical analyses based on datasets with missing values introduce biases, emphasizing the need for interventions to enhance data completeness within quality assurance programs. Accurate data registration not only facilitates prospective scientific purposes but also exploits the value data reuse [31]. However, it should be recognized that completeness alone does not guarantee data quality. Aspects like consistency (avoiding discrepancies in comparing data items), timeliness, accuracy, uniqueness, and validity must also be addressed for optimal data quality [18,38].

From a scientific perspective, it is important to recognize that data completeness alone does not guarantee quality; aspects like consistency, timeliness, accuracy, and validity must also be addressed. Although these aspects were beyond the scope of this study, they are essential in quality assessment programs. Structured data entry within electronic health records facilitates data-driven healthcare. While manual data checks provide partial oversight, automated monitoring and alerting, utilizing data observability [39] and root-cause analysis tools, could enable proactive management of data quality. Manual data collection is initially a time-consuming exhibition and is nowadays often taken for granted, while automated data collection saves time for healthcare professionals.

Recent studies have shown that structured settings allow up to 99% agreement between manual and automated data collection [31]. Artificial intelligence could also enhance data quality by automating data generation and addressing challenges associated with big data [40]. AI’s reliance on data accuracy highlights the importance of healthcare professionals’ awareness of their contribution to data integrity, ultimately benefiting data quality. This study’s focus on implementing a data-driven dashboard feedback tool not only aims to improve data completeness but also supports overall data quality in our healthcare setting [41].

While promising, this study focuses on data completeness alone and does not directly address whether this improvement translates to better overall data quality or quality of care. The literature suggests that more complete data can lead to better-informed treatment decisions, but additional steps are necessary to confirm this. We plan to conduct a follow-up analysis to assess the dashboard’s long-term impact on data completeness and to examine how completeness is reflected in clinical decisions, such as family history considerations or earlier biopsy initiation. These findings will be presented in a separate publication.

Finally, quality assurance programs are no stand-alone “research” projects. They are continuous cycles of improvement incorporated into daily practice to ensure the best possible care. While the quality cycles warrant the quality of care, participating healthcare professionals should warrant the quality of the cycle. Quality assurance is achieved through the collection and analysis of reliable data and the willingness of healthcare professionals to act on these findings. To fulfill improvement of data entry, the combination of progressing prospective registry databases and collaboration with a data scientist is essential. This collaborative approach allows for the development of robust data collection methods and analyses, ensuring the accuracy and completeness of data collected. By leveraging the expertise of data scientists, healthcare professionals can gain valuable insight into their data, leading to informed decision making and enhanced patient care.

While this study provides an initial evaluation of the dashboard’s impact, the results highlight the need for continuous quality assurance. Quality assurance programs are not isolated efforts; they require sustained engagement by healthcare professionals to adapt to evolving standards and ensure reliable data collection. To achieve meaningful improvements, ongoing collaboration with data scientists is essential, allowing healthcare professionals to leverage data insights to improve patient care.

## 5. Conclusions

The data-driven dashboard significantly improved data completeness in the early detection pathway for prostate cancer. This enhancement supports the potential of dashboards to aid in diagnostic workflows, clinical decision making, and foster a data-driven approach to healthcare. Long-term results are needed to definitively confirm these results. Involving healthcare professionals in the development and implementation of a data-driven dashboard feedback tool is crucial to improve data quality within quality assurance programs.

## Figures and Tables

**Table 1:** Descriptive table listing all 10 items included in the questionnaire, organized by their corresponding domains. The questionnaire used a 5-point Likert-type agree–disagree scale and includes responses from all eight healthcare providers (response rate: 100%). Results are displayed as medians, with the interquartile range (IQR) shown in parentheses.

Item	Domain	Questions All Started with ‘From Your Perspective:…’	Median Score on 5-Point Likert Scale (IQR)
1.	Usefulness	The dashboard visualized a clear overview of data items	4 (3.5–4.5)
2.	Usefulness	The dashboard was simple and easy to understand	4 (3.5–5)
3.	Interface	The dashboard interface was pleasant	4 (4–5)
4.	Interface	You liked using the functions of the dashboard	4 (4–4)
5.	Reliability	You thought that the dashboard reports were reliable	4 (3–4)
6.	Reliability	You thought that the dashboard correctly presented the missing data items	4 (3–4.5)
7.	Effectiveness	The dashboard motivated you to register all data items	5 (4–5)
8.	Effectiveness	The dashboard improved your awareness of data registry	5 (4.3–5)
9.	Adaption	Weekly receiving the dashboard was a good frequency	5 (3–5)
10.	Adaption	I thought it was easily integrated in routine work	4 (4–5)

**Table 2:** Overview of the literature on the utilization of dashboards in healthcare settings.

Author	Investigator	Title	Source	Summary of Findings
Twohig, P.A. et al. (2019)	[21]	Clinician dashboard views and improvement in preventative health outcome measures: a retrospective analysis	BMC Health Services Research	Clinical dashboard on their own may not be sufficient to impact clinical quality improvement.
Dowding, D. et al. (2015)	[22]	Dashboards for improving patient care: review of the literature	International Journal of Medical Informatics	Implementation of clinical and/or quality dashboards can improve adherence to quality guidelines and may help improve patient outcomes.
Gude, W.T. et al. (2018)	[23]	Health professionals’ perceptions about their clinical performance and the influence of audit and feedback on their intentions to improve practice: a theory-based study in Dutch intensive care units	Implementation Science	Audit and feedback help health professionals to work on aspects for which improvement is recommended.
Murphy, D.R. et al. (2021)	[24]	Dashboards for visual display of patient safety data: a systematic review	BMJ Health Care Informatics	Use of patient safety dashboards has grown over the past 15 years, but impact remains poorly understood. Design and usability evaluation of patient safety dashboards should incorporate informatics and human factors principles.
Wac, M. et al. (2023)	[25]	Design and evaluation of an intensive care unit dashboard built in response to the COVID-19 pandemic: semi-structured interview study	JMIR human factors	Implementation of a dashboard had a positive impact on the workflows, improving efficient patient care and transformed existing processes.
Perry, L.M. et al. (2022)	[26]	Patient-reported outcome dashboards within the electronic health record to support shared decision making: protocol for co-design and clinical evaluation with patients with advanced cancer and chronic kidney disease	JMIR Research Protocols	Enhancing current understanding of how best to integrate patient-reported outcome measures in dashboards to improve outcomes and reduce burden of chronic disease.
Elm, JJ. et al. (2019)	[27]	Feasibility and utility of a clinician dashboard from wearable and mobile application Parkinson’s disease data	NPJ Digital Medicine	Clinicians reported that medications and patient-reported outcomes were generally discernable in the dashboard and complementary to clinical assessments.
Durojaiye, A.B. et al. (2018)	[28]	Radiology resident assessment and feedback dashboard radiographics	Radiographics	Describing the dashboard’s architecture to provide residents real-time feedback in a learning community of radiologists.
Baghdadi, A. et al. (2021)	[29]	A data-driven performance dashboard for surgical dissection	Scientific reports	A surgical performance and monitoring data interactive interface to develop artificial intelligence models.
McCormick, C., S. Ahluwalia, and A. Segon (2023)	[30]	Effect of a performance feedback dashboard on hospitalist laboratory test utilization	American Journal of Medical Quality	Integrating a weekly performance feedback report using a dashboard resulted in a decrease in laboratory test utilization rates.
Ebbers, T. et al. (2023)	[31]	Development and validation of automated electronic health record data reuse for a multidisciplinary quality dashboard	Digital health	The possibility to use routinely collected structured data to reliably measure the quality of care in real time, which could render manual data collection for quality measurement obsolete.
Lechner, C. et al. (2022)	[32]	Developing an online dashboard to visualize performance data—Tennessee newborn screening experience	International Journal Neonatal screening	Performance data of newborn screening process captured in a dashboard at state level, identifying potential issues.
Tsangaris, E. et al. (2022)	[33]	User-centered design and agile development of a novel mobile health application and clinician dashboard to support the collection and reporting of patient-reported outcomes for breast cancer care	BMJ surgical intervention health technology	Utilization of dashboard to collect patient-reported outcomes in breast cancer care and enhance digital transformation.
Mohindra, N.E. et al. (2024)	[34]	Implementing a patient-reported outcome dashboard in oncology telemedicine encounters: clinician and patient adoption and acceptability	JCO oncology practicre	Feasible implementation of a patient-reported outcome-based dashboard in telemedicine.
Alhmoud, B. et al. (2022)	[35]	Evaluating a novel, integrative dashboard for health professionals’ performance in managing deteriorating patients: a quality improvement project	BMJ open quality	Dashboard demonstrated to be an effective real-time data-driven tool for improving the quality of managing deteriorating patients.

**Table 3:** Baseline characteristics of patients enrolled in the early detection pathway for prostate cancer.

Patient Characteristics	Outcome Measures
Age Years Median (IQR)	67 (61–73)
Psa ng/mL Median (IQR)	7.9 (4.7–12.4)
Direct Rectal Exam, N (%)	577 (100%)
Abnormal	230 (39.9%)
Normal	347 (60.1%)
Prostate Mri, PI-RADS, N (%)	577 (100%)
1	3 (0.5%)
2	121 (20%)
3	99 (17.2%)
4	133 (23.1%)
5	211 (36.6%)
Prostate Biopsies Performed in N (%)	389 (67.4%)
Isup Grade N (%)	304 (100%)
1	74 (24.3%)
2	120 (39.5%)
3	62 (20.4%)
4	25 (8.2%)
5	23 (7.6%)

## Data Availability

The data presented in this study are available on request from the corresponding author. The data are not publicly available due to privacy reasons.

## Supplementary Materials

The following supporting information can be downloaded at: https://www.mdpi.com/article/10.3390/jcm13247529/s1.
