# Supplementary material for: Enhancing Data Completeness in Early Detection Pathway of Prostate Cancer: Integration of a Dashboard-Driven Feedback Tool to Improve Quality of Care
Source: J Clin Med. 2024 Dec 11;13(24):7529. doi: 10.3390/jcm13247529 (PMC11728300; doi:10.3390/jcm13247529)
Supplement: Supplementary file 1 [file jcm-13-07529-s001.zip › jcm-3236276-supplementary.pdf]

Supplementary

Table S1. Overview of all stations of the dashboard connected with the electronic health records.

	**kaiko_station**	**question_description**
**0**	Biopsy	Prostate biopsies—MRI left
**1**	Biopsy	Prostate biopsies—MRI right
**2**	Biopsy	Prostate biopsies—rectal direct examination left
**3**	Biopsy	Prostate biopsies—rectal direct examination right
**4**	Biopsy	Prostate biopsies—zone 1
**5**	Biopsy	Prostate biopsies—zone 2
**6**	Biopsy	Prostate biopsies—zone 3
**7**	Biopsy	Prostate biopsies—zone 4
**8**	Biopsy	Prostate biopsies—zone 5
**9**	Biopsy	Prostate biopsies—zone 6
**10**	biopsy_consult	Target zone 1 a-b
**11**	biopsy_consult	Target zone 1 c-d
**12**	biopsy_consult	Target zone 2 a-b
**13**	biopsy_consult	Target zone 2 c-d
**14**	biopsy_consult	Target zone 3 a-b
**15**	biopsy_consult	Target zone 3 c-d
**16**	biopsy_consult	Target zone 4 a-b
**17**	biopsy_consult	Target zone 4 c-d
**18**	biopsy_consult	Target zone 5 a-b
**19**	biopsy_consult	Target zone 5 c-d
**20**	biopsy_consult	Target zone 6 a-b
**21**	biopsy_consult	Target zone 6 c-d
**22**	first_consult	Definitive treatment conclusion
**23**	first_consult	Previously negative prostate biopsies
**24**	first_consult	Mamma carcinoma diagnosis with age under 50 years
**25**	first_consult	Ovarian carcinoma
**26**	first_consult	Pancreas carcinoma
**27**	first_consult	Prostate volume TRUS (cc)
**28**	first_consult	Prostate cancer (Gleason ≥7) in 3 executive family members
**29**	first_consult	Prostate cancer (Gleason ≥7) before the age of 50 years
**30**	first_consult	Risk calculation for significant prostate cancer
**31**	first_consult	Risk calculation for prostate cancer
**32**	first_consult	Triage: Rectal direct examination left
**33**	first_consult	Triage: Rectal direct examination right
**34**	first_consult	Contact person
**35**	first_consult	≥ 2 direct family members with prostate cancer ((Gleason ≥7)
**36**	follow_up	PSA
**37**	mdo_biopsy	Positive biopsies left
**38**	mdo_biopsy	Positive biopsies right

**39**	mdo_biopsy	Biopsies left
**40**	mdo_biopsy	Biopsies right
**41**	mdo_biopsy	Charlson probability
**42**	mdo_biopsy	Continent prediction if no nerve sparing
**43**	mdo_biopsy	Cribriform growth
**44**	mdo_biopsy	Highest Gleasonscore left
**45**	mdo_biopsy	Highest Gleasonscore right
**46**	mdo_biopsy	Local treatment options
**47**	mdo_biopsy	MTD prostate: Prostate volume (cc)
**48**	mdo_biopsy	PIRADS
**49**	mdo_biopsy	PSA
**50**	mdo_biopsy	Pathology biopsies
**51**	mdo_biopsy	Percentage left
**52**	mdo_biopsy	Percentage right
**53**	mdo_biopsy	Type TNM
**54**	mdo_biopsy	Pathway prostate
**55**	mdo_mri	MTD Prostate: Prostate volume (cc)
**56**	mdo_mri	PIRADS
**57**	mdo_mri	PSA
**58**	mdo_mri	Prostate volume TRUS (cc)
**59**	mdo_mri	Risk calculation for significant prostate cancer
**60**	mdo_mri	Risk calculation for prostate cancer
**61**	mdo_mri	Triage: Rectal direct examination left
**62**	mdo_mri	Triage: Rectal direct examination right
**63**	mdo_mri	Pathway prostate
**64**	mri_consult	Treatment option after MRI
**65**	mri_consult	MTD Prostate: Prostate volume (cc)
**66**	mri_consult	PIRADS
**67**	mri_consult	PSA
**68**	mri_consult	Risk calculation for significant prostate cancer
**69**	mri_consult	Risk calculation for prostate cancer
**70**	Triage	Abnormal PSA early detection of prostate pathway
**71**	video_consult	Treatment option after MRI
**72**	video_consult	Conclusion family history prostate carcinoma
**73**	video_consult	Previously negative prostate biopsies
**74**	video_consult	IIEF-5 score
**75**	video_consult	IPSS score
**76**	video_consult	MTD Prostate: Prostate volume (cc)
**77**	video_consult	Mamma carcinoma diagnosis with age under 50 years…
**78**	video_consult	Ovarian carcinoma
**79**	video_consult	PI-RADS
**80**	video_consult	PSA
**81**	video_consult	Pancreas carcinoma

**82**	video_consult	Prostate carcinoma (Gleason ≥7) in 3 executive family members
**83**	video_consult	Prostate carcinoma (Gleason ≥7) before the age of 50 years
**84**	video_consult	Risk calculation for significant prostate cancer
**85**	video_consult	Risk calculation for prostate cancer
**86**	video_consult	≥ 2 direct family members with prostate cancer (Gleason ≥7)
